# Extranodal natural killer/T-cell lymphoma in Malawi: a report of three cases

**DOI:** 10.1186/s12885-017-3612-y

**Published:** 2017-09-06

**Authors:** Tamiwe Tomoka, Eric Powers, Toon van der Gronde, Amy Amuquandoh, Bal Mukunda Dhungel, Coxcilly Kampani, Steve Kamiza, Nathan D. Montgomery, Yuri Fedoriw, Satish Gopal

**Affiliations:** 1 UNC Project-Malawi, Private Bag A104, Lilongwe, Malawi; 2 University of North Carolina, Chapel Hill, USA; 3 University of Malawi College of Medicine, Blantyre, Malawi

**Keywords:** Non-Hodgkin lymphoma, Epstein-Barr virus, Sub-Saharan Africa

## Abstract

**Background:**

Extranodal NK/T-cell lymphoma (ENKTCL) reports from sub-Saharan Africa (SSA) are remarkably rare, despite early childhood acquisition and high prevalence of the causative infectious agent, Epstein-Barr virus (EBV), and frequent occurrence of other lymphoproliferative disorders causally associated with EBV.

**Case presentations:**

At a national teaching hospital in Malawi, three patients of African descent were seen with ENKTCL between 2013 and 2014. Patients were aged between 29 and 60 years, two with craniofacial involvement and one with a primary abdominal tumor, and all were HIV-negative. All had systemic B symptoms, and two severely impaired performance status. On histologic review, morphology and immunophenotyping demonstrated classical ENKTCL features in all cases, including diffuse proliferations of intermediate-to-large atypical lymphocytes with high mitotic activity and extensive background necrosis, positivity for CD3 and CD56, and negativity for CD20. By in situ hybridization, all three tumors were positive for EBV-encoded RNA (EBER). Baseline plasma EBV DNA was also markedly elevated for all three patients. Due to radiotherapy and chemotherapy limitations, patients were treated with CHOP (cyclophosphamide, doxorubicin, vincristine, prednisone) with rapid disease progression. All three patients died from progressive lymphoma within 3 months of initial diagnosis.

**Conclusions:**

Our experience with these three patients in Malawi can highlight that ENKTCL does indeed occur in SSA, increase familiarity with ENKTCL among clinicians and pathologists throughout the region, and emphasize the need for better diagnosis and treatment for this neglected population.

## Background

It is vital to improve lymphoma diagnostic infrastructure in sub-Saharan Africa (SSA) to fully understand the heterogeneity of non-Hodgkin lymphoma (NHL) in the region, provide better prognostic information to patients and clinicians, and allow more subtype-directed therapy where appropriate. In resource-rich settings, molecular profiling is increasingly leading to tailored therapeutic approaches even within well-defined histologic categories, like diffuse large B-cell lymphoma. However, due to resource limitations in SSA, the entire spectrum of NHL is often diagnosed and treated as a single entity. Among low- and middle-income countries, extranodal NK/T-cell lymphoma (ENKTCL) in particular has been principally described in Asia and Latin America, and published reports from SSA are remarkably few. This is despite early childhood acquisition and high prevalence of the causative infectious agent, Epstein-Barr virus (EBV), in SSA, as well as frequent occurrence of other lymphoproliferative disorders that are causally associated with EBV, like endemic Burkitt lymphoma and classical Hodgkin lymphoma. Therefore, to increase regional awareness of this disease, highlighting the occurrence of ENKTCL specifically in SSA is important.

**Fig. 1:**
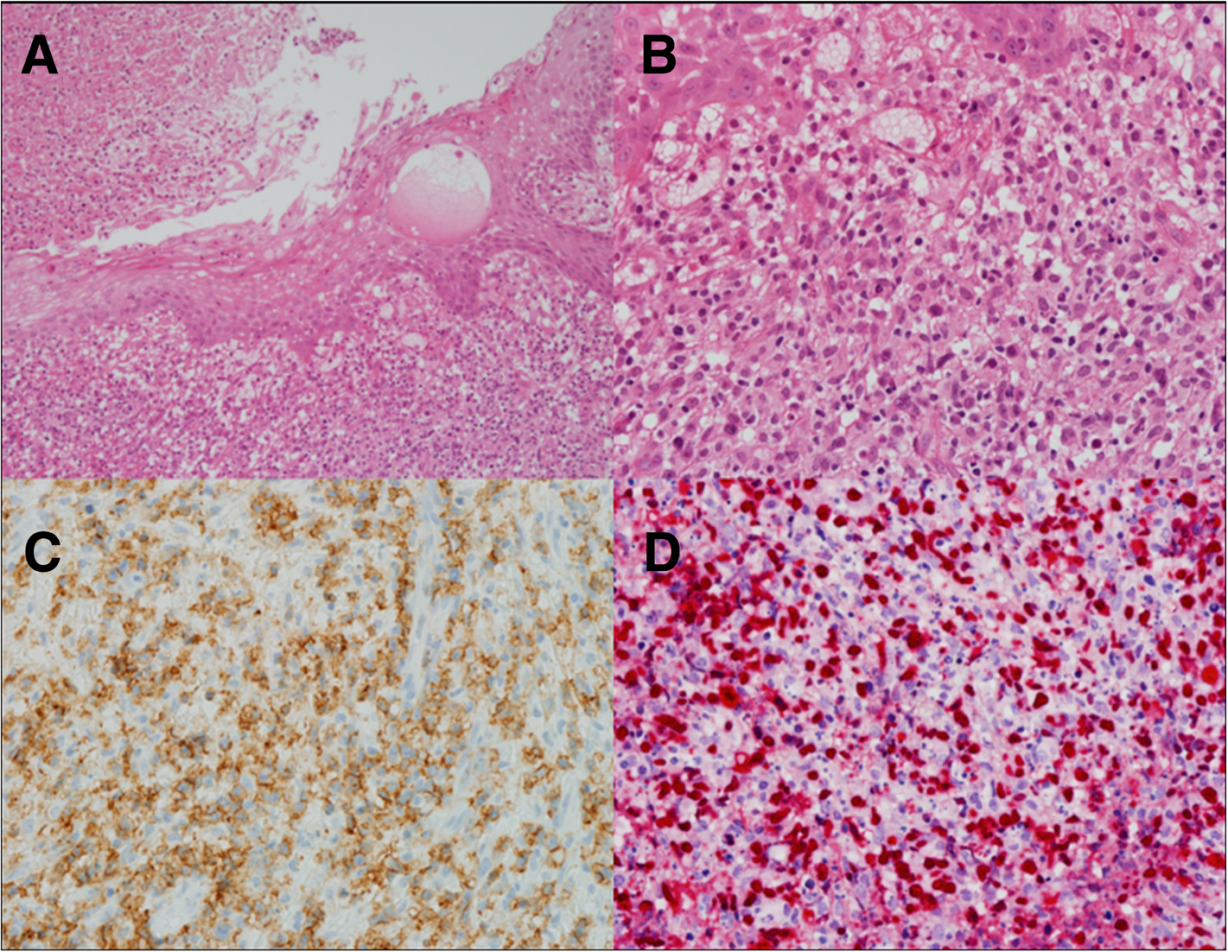
Biopsy of sinonasal mass from Case 2. **a** Ulcerated squamous mucosa surrounded by necrosis and subepithelial proliferation of tumor cells (haematoxylin and eosin, 10×). **b** Moderate-to-large atypical lymphoid-appearing tumor cells with high mitotic activity (haematoxylin and eosin, 40×). **c** Tumor cells positive for CD56 by immunohistochemistry (40×). **d** Tumor cells positive for Epstein-Barr virus-encoded RNA by in situ hybridization (40×)

## Case presentations

At a national teaching hospital in the capital of Malawi, Lilongwe, three patients of African descent were seen with ENKTCL between September 2013 and March 2014, and were enrolled in the prospective Kamuzu Central Hospital Lymphoma Study cohort after informed consent. Case 1 was a 29-year-old male with a greater than 6 month history of an abdominal mass. Case 2 was a 51-year-old male who presented with a 4 month history of a sinonasal tumor eroding the medial wall of the maxilla associated with right eye proptosis. Case 3 was a 60-year-old female with a less than 3 month history of progressive left maxillary sinus mass involving the medial left and right orbits. All three patients were HIV-negative, all had systemic B symptoms, and two had severely impaired Eastern Cooperative Oncology Group performance status of greater than 2.

Biopsy and tumor characterization using immunohistochemistry performed locally in Lilongwe was done for all patients, and was reviewed in real time during a weekly telepathology conference conducted between clinicians and pathologists in Lilongwe and Chapel Hill, as previously described in detail [1]. Histologically, all three cases showed diffuse proliferations of intermediate-to-large atypical lymphocytes with high mitotic activity and areas of extensive background necrosis, as shown for a single case exhibiting features typical of all three in the accompanying Fig. 1. Immunophenotypically, the tumors were all positive for CD3 and CD56, and negative for CD20. Subsequent in situ hybridization (ISH) in Chapel Hill demonstrated all three tumors to be positive for EBV-encoded RNA (EBER). All patients had staging bone marrow evaluations without involvement by lymphoma.

Other baseline laboratory investigations were normal excepting mild anemia (hemoglobin 10.8 g/dL) in one case, and elevated lactate dehydrogenase in two cases. Baseline plasma EBV DNA was also markedly elevated for all three patients by real-time quantitative polymerase chain reaction, ranging from 3412 to 2,098,940 copies/mL. Radiotherapy is currently not available anywhere in Malawi, and limited supportive care infrastructure and low drug availability prohibit intensive chemotherapy regimens including asparaginase or high-dose methotrexate. Therefore, the local standard of care for most NHL subtypes is CHOP (cyclophosphamide, doxorubicin, vincristine, prednisone). Of our three patients, one was too sick to initiate cytototoxic treatment and two received a single dose of CHOP with rapid subsequent disease progression. All three patients died from progressive lymphoma within 3 months of initial diagnosis.

## Discussion and conclusions

ENKTCL is an uncommon subtype of the the mature T and NK-cell neoplasms, a heterogeneous group of aggressive NHL that account for approximately 12% of all NHL in the United States, Europe, and Asia [2]. ENKTCL typically involves the upper aerodigestive tract, with the nasal cavity being the prototypic site of involvement. Sites for extranasal involvement include skin, soft tissue, gastrointestinal tract, and testes [3]. Our cases highlight the rapid clinical progression of this disease and short overall survival, particularly after suboptimal treatment as in our setting. Importantly, CHOP was administered in our setting due to lack of more suitable alternatives like aparaginase or high-dose methotrexate, and poor outcomes after CHOP for ENKTCL are have been attributed to tumor expression of multidrug resistance protein [4].

There is significant ethnic and regional variation in ENKTL prevalence worldwide. It is most common in East and Southeast Asia, as well as regions of Central and South America, where it has been reported to represent 6–15% of NHL cases [5–7]. Frequency in SSA and persons of African descent is not extensively described, but is presumed to be low, although this may reflect under-recognition and under-diagnosis given well documented diagnostic limitations in the region. Retrospective epidemiologic studies in the United States have found non-Hispanic black persons to account for 2 to 5% of all ENKTCL cases [8]. There are also a very small number of published reports of ENKTCL in SSA [9–11], in addition to other studies describing NHL of the nasal cavity and nasopharynx without complete histopathologic or immunophenotypic characterization [12, 13].

Pathologically, ENKTCL most commonly expresses an NK cell phenotype, being positive for CD56, cytoplasmic CD3ε+, without surface T-cell receptor (TCR) and TCR gene rearrangements [2, 14]. A subset of cases may demonstrate a T-cell phenotype with clonal TCR gene rearrangements. ENKTCL is strongly associated with EBV, which can be uniformly demonstrated in tumors by EBER ISH and plasma by qPCR [15, 16]. Plasma EBV DNA is correlated with other markers of disease severity like clinical stage and serum lactate dehydrogenase. Plasma EBV DNA levels at baseline are also associated with clinical outcomes, and serial measurement may be valuable for assessing response to treatment [16].

In conclusion, although EBV is highly prevalent in SSA with acquisition typically during early childhood, and other EBV-associated lymphoproliferative disorders including endemic Burkitt lymphoma and classical Hodgkin lymphoma are often described, ENKTCL reports from SSA are scarce, likely reflecting under-diagnosis. We hope our experience with these three patients in Malawi can serve to highlight that ENKTCL does indeed occur in SSA, increase familiarity with ENKTCL among clinicians and pathologists throughout the region, and emphasize the need for better diagnosis and treatment for this neglected population.

## Abbreviations

CHOP: Cyclophosphamide, doxorubicin, vincristine, prednisone;
EBER: Epstein-Barr virus-encoded RNA
EBV: Epstein-Barr virus
ENKTCL: Extranodal NK/T-cell lymphoma
NHL: Non-Hodgkin lymphoma
SSA: Sub-Saharan Africa
